# Missed Intraocular Foreign Body Presenting As Siderosis Bulbi: Two Case Reports

**DOI:** 10.7759/cureus.53839

**Published:** 2024-02-08

**Authors:** Chuah Gim Seah, Julieana Muhammed, Lee Annie, Khairuddin Othman

**Affiliations:** 1 Department of Ophthalmology, Hospital Sultanah Bahiyah, Alor Setar, MYS; 2 Department of Ophthalmology and Visual Science, School of Medical Sciences, Universiti Sains Malaysia, Kubang Kerian, MYS

**Keywords:** ocular trauma, pars plan vitrectomy, computed tomography, missed intraocular foreign body, siderosis bulbi

## Abstract

Ocular siderosis bulbi is a rare but significant cause of vision impairment in patients with a retained ferrous intraocular foreign body (IOFB). In this report, we present two cases of ocular manifestations suggestive of siderosis bulbi. Both cases presented with a significant reduction in vision and were found to have a dense cataract, a small healed corneal scar, and siderotic pigments in the anterior chamber. The first case denied any past ocular injury, yet CT scans confirmed the presence of an IOFB. The second case, who had a high suspicion of ocular trauma, did not have a radiologically detectable IOFB. Both cases underwent combined cataract extraction surgery with pars plana vitrectomy and IOFB removal, resulting in a favorable visual outcome despite developing siderosis.

## Introduction

Intraocular foreign bodies (IOFBs) can manifest insidiously, accounting for 18%-41% of all open globe injuries [1]. Failure to detect them can lead to devastating consequences [2]. In this report, we present two cases of missed IOFBs, both presenting as clinical ocular siderosis bulbi.

Siderosis bulbi is defined as pigmentary and degenerative changes in the eye resulting from the retention of iron-containing IOFBs [3]. Computed tomography (CT) and ultrasonography are considered the gold standard for the early detection of IOFBs [4]. However, our second case presented with clinical ocular siderosis, yet no detectable IOFB was found on CT and ultrasonography. Misdiagnosis is a risk when dealing with an indefinite, radiologically undetectable IOFB [5].

These cases underscore the importance of taking a careful history and conducting a detailed ocular assessment, especially with a high index of suspicion for patients presenting with visual impairment, particularly those working in industrial workplaces and of working age. We discuss the devastating sequelae of a missed retained IOFB (siderosis bulbi) and the positive vision outcome following its removal [6]. Both cases have provided informed consent for the publication in the journal. The first case was previously presented as a conference poster at the 15th Congress of the Asia-Pacific Vitreo-Retina Society on November 18-20, 2022.

## Case presentation

Case 1

A 38-year-old gentleman, who works as a construction worker, presented with a four-month history of progressive, painless deterioration of vision in his right eye in July 2022. On careful questioning, he mentioned feeling some foreign body sensation over the right eye but denied any previous eye injury. Upon examination, the vision in the right eye was 1/60, while the left eye had a vision of 6/6.

**Figure 1:**
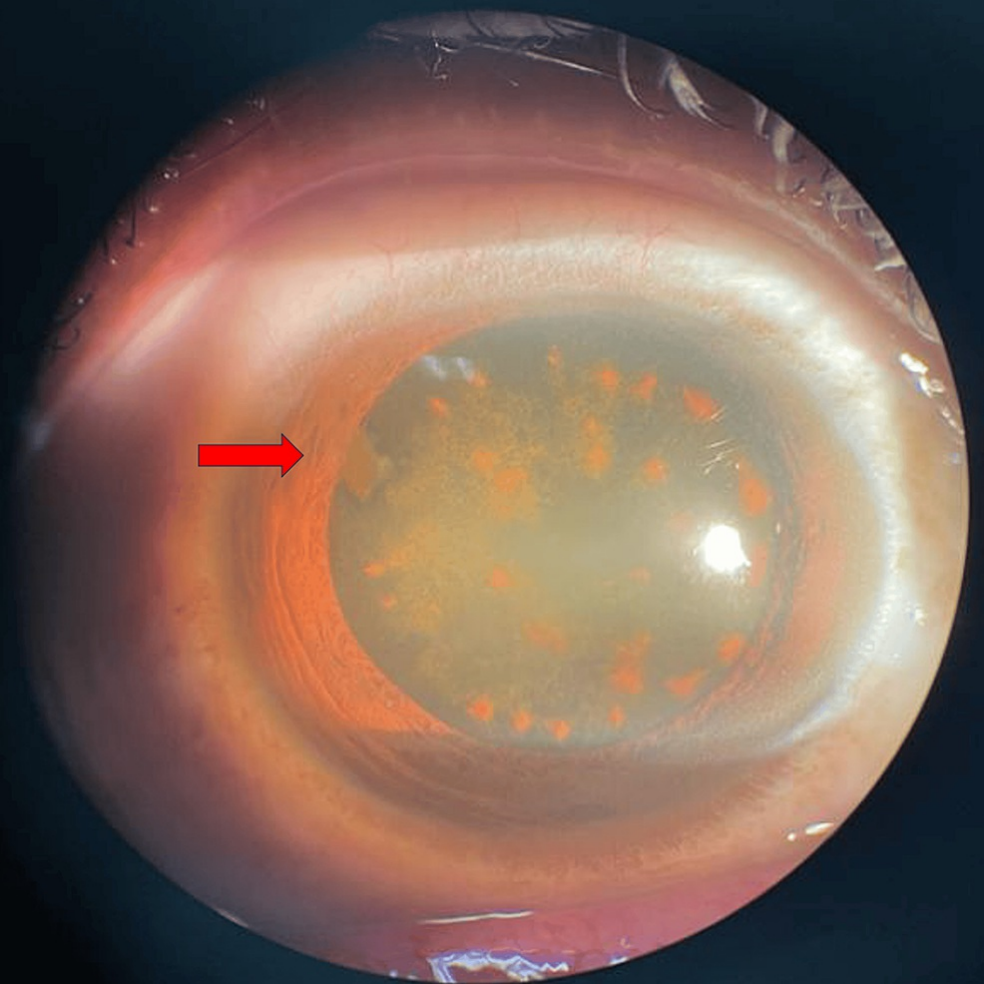
The right eye exhibited a supero-temporal, self-sealed corneal scar, with siderotic pigments on the cataractous lens.

A slit lamp examination of the right eye revealed an oblique, self-sealed old corneal wound in the supero-temporal region, accompanied by a corresponding iris defect beneath the corneal wound. The cornea appeared hazy with intrastromal rust-colored deposition, and siderotic pigments were observed in the anterior chamber. The pupil was dilated with a yellowish cataract and brown deposits on the capsule (Figure 1). Normal intraocular pressure was measured at 14mmHg using a Goldmann applanation tonometer. Fundoscopy was impractical in the right eye. A B-scan ultrasound showed a silent vitreous and flat retina.

**Figure 2:**
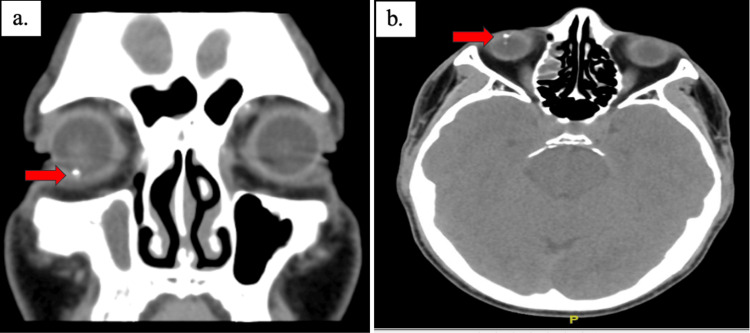
Coronal view of plain computed tomography of the orbit showing a hyperdense focus seen at the inferior aspect (a); axial view showing two hyperdense foci at the inferior aspect of the posterior segment, suggestive of intraocular foreign bodies (IOFB) in the right globe (b).

A plain CT of the orbit was performed; the coronal view showed a hyperdense focus at the inferior aspect of the posterior segment of the right globe (Figure 2a), but the axial view showed two hyperdense foci at the inferior aspect of the posterior segment, suggestive of IOFBs measuring 2.6mm and 2.5mm, respectively (Figure 2b). He underwent plain cataract extraction with pars plana vitrectomy. Only one metallic foreign body was found embedded in the pars plana. The two hyperdense foci seen on the CT scan were probably the two ends of the foreign body. No retinal breaks were observed, and the retina was flat.

One month postoperatively, the best-corrected visual acuity (BCVA) was 6/9 with a refraction of +13.00/-0.75 x 145 in the right eye and -0.25DS in the left eye.

Case 2

A 30-year-old gentleman who works as a fisherman presented in February 2022 with painless, gradual deterioration of vision in his right eye for the past six months. He reported a history of hammering months before the onset of symptoms and felt a foreign body sensation in his right eye but did not seek any treatment. Upon presentation, the right eye had a visual acuity of 6/24, and the left eye had a visual acuity of 6/9. There was no relative afferent pupillary defect (RAPD) observed.

**Figure 3:**
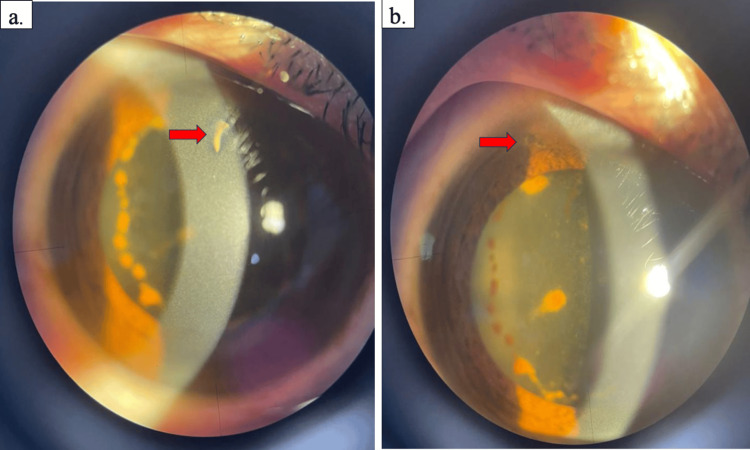
The right eye revealed a self-sealed shelving corneal laceration wound at the paracentral 12 o’clock position (a), along with a corresponding iris defect beneath the corneal wound (b).

Slit lamp examination of the right eye revealed a self-sealed, shelving corneal laceration wound at the paracentral 12 o’clock position, measuring around 1.5mm in length vertically (Figure 3a), along with a corresponding iris defect beneath the corneal wound (Figure 3b). The cornea appeared hazy with a significant amount of siderotic pigments on the endothelium, but Seidel’s test was negative for any active leakage. The anterior chamber was deep with occasional cells and showed a yellowish tinge of aqueous humor. Pupils were mid-dilated, and the crystalline lens exhibited cataractous changes with partial anterior capsular fibrosis and multiple clumps of pigments in a circular shape from 1 to 2 o’clock. Normal intraocular pressure was measured at 16mmHg using a Goldmann applanation tonometer. Fundus examination showed a hazy view with the optic disc (OD) appearing pink and a cup-to-disc ratio (CDR) of 0.3, with a flat retina but no IOFB noticeable.

**Figure 4:**
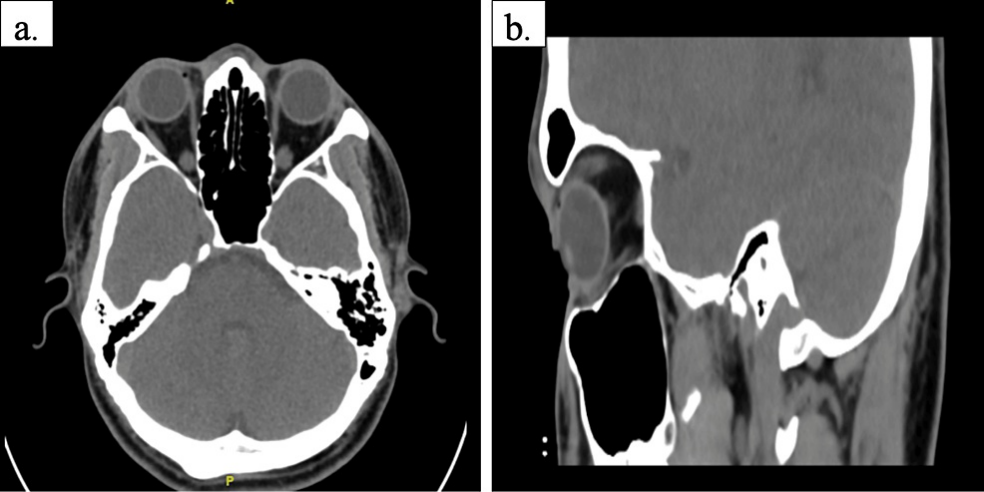
Plain computed tomography of the orbit showed no detectable intraocular radiopaque foreign body in both axial (a) and sagittal (b) views.

No IOFB was detected on the B-scan, and urgent plain computed tomography of the orbit (Figure 4) was performed.

After four months, the patient’s right eye vision deteriorated further to counting fingers, with worsening of the cataractous lens. He then underwent right eye plain cataract extraction surgery with combined pars plana vitrectomy. One metallic, flat foreign body was found lodged anterior to the ora serrata, but no retinal breaks were observed, with the retina appearing flat. One month post-operatively, the visual acuity in the right eye improved to 6/9 with a +10 lens.

## Discussion

Siderosis is a distinctive clinical presentation resulting from the presence of iron-contaminated intraocular tissues. This condition affects various ocular structures, with notable changes observed in the iris and lens, leading to iris heterochromia (58.33%) and cataract formation (95.83%) [7]. Additionally, the retina may undergo pigmentation degeneration (58.33%) [4,7,8].

One of the hallmark indicators of early siderosis is the development of a cataract (95.83%) [7], which can be a sensitive signal of iron deposition. Lens siderosis involves the accumulation of iron in the epithelial cells of the anterior capsule, resulting in numerous brownish or rusty spots. As the condition progresses, the entire lens may acquire a deep yellow hue with large, rusty-brown patches [2,4].

Unfortunately, the toxic changes associated with siderosis become irreversible beyond a certain stage. Therefore, early detection with surgical removal of the metallic foreign body is crucial treatment for almost all patients. This intervention aims to prevent further iron-related damage and preserve ocular function [4].

While most cases of siderosis bulbi are associated with a definite history of ocular injury, our first case denied any such history. However, there must be a high suspicion in patients working industrial jobs, as this serves as a high risk for ocular injury. In our case, the IOFB was likely small, with a low concentration of iron-containing metallic alloy. It was sequestered in the pars plana, masking the early presentation of ocular inflammation [6].

Computed tomography (CT) is the gold standard for the detection of an occult foreign body [4]. The detection of small foreign bodies on CT relies on the CT scanner’s resolution capability and the foreign body’s density, size, and position. In the second case, despite a history of ocular trauma and clinical presentation highly suspicious of siderosis bulbi, no solid proof of an IOFB was verified on ultrasound B-scan and CT scan. This could be attributed to the small size of the IOFB, potentially missed by conventional contrast-enhanced CT technique [4,9]. Notably, our CT unit produced contiguous 2-mm images that might have affected the detection of a very small foreign body.

B-scan can be helpful in detecting the IOFB, but its effectiveness relies on the operator’s skill, highlighting the operator’s role in performing the scan. Additionally, the chronic retention of the iron may contribute to the lack of radiological detection as the iron could undergo dissolution over time [9].

Siderosis bulbi, without a radiologically detected foreign body, is a rare condition that has been reported as a result of intrascleral rust, intracorneal, and intralenticular residue [4,5,9].

Iron retinotoxicity can cause dysfunction across all layers of the retina, and this damage may be reversible during the early stages of the disease. Electrophysiology is a valuable tool for assessing retinal function and the extent of any toxic retinal damage [8]. However, in our center, this evaluation was not performed due to the unavailability of the required device. This serves as a limitation for our case report.

Pars plana vitrectomy is the preferred treatment modality for the removal of IOFBs. An early approach to IOFB removal prevents vision-threatening complications such as endophthalmitis and siderosis [8].

## Conclusions

In conclusion, the two cases demonstrate the devastating sequelae that can result from missed IOFBs. Patients’ lack of awareness regarding ocular trauma, coupled with delayed medical attention, was a common cause of siderosis bulbi with retained IOFBs. These cases emphasize the importance of conducting a detailed investigation for any condition involving worsening visual acuity, especially in the working-age population and those in certain occupational fields where penetrating injuries causing retention of IOFB are likely. It is also crucial to raise suspicion in patients exhibiting ocular manifestations of siderosis bulbi, even in the absence of a definitively radiologically detectable IOFB. Prompt diagnosis and early management are crucial to achieving a favorable visual outcome.
